# GRHL2-controlled gene expression networks in luminal breast cancer

**DOI:** 10.1186/s12964-022-01029-5

**Published:** 2023-01-23

**Authors:** Zi Wang, Bircan Coban, Haoyu Wu, Jihed Chouaref, Lucia Daxinger, Michelle T. Paulsen, Mats Ljungman, Marcel Smid, John W. M. Martens, Erik H. J. Danen

**Affiliations:** 1 Leiden Academic Center for Drug Research, Leiden University, Leiden, The Netherlands; 2 Department of Human Genetics, Leiden University Medical Centre, Leiden, The Netherlands; 3 Departments of Radiation Oncology and Environmental Health Sciences, University of Michigan Medical School, Ann Arbor, MI USA; 4 Department of Medical Oncology, Erasmus MC Cancer Institute, Erasmus University Medical Center, Rotterdam, The Netherlands

**Keywords:** Breast cancer, Luminal, GRHL2, CHIP-seq, BRU-seq, Transcription, Gene regulation

## Abstract

Grainyhead like 2 (GRHL2) is an essential transcription factor for development and function of epithelial tissues. It has dual roles in cancer by supporting tumor growth while suppressing epithelial to mesenchymal transitions (EMT). GRHL2 cooperates with androgen and estrogen receptors (ER) to regulate gene expression. We explore genome wide GRHL2 binding sites conserved in three ER⍺/GRHL2 positive luminal breast cancer cell lines by ChIP-Seq. Interaction with the ER⍺/FOXA1/GATA3 complex is observed, however, only for a minor fraction of conserved GRHL2 peaks. We determine genome wide transcriptional dynamics in response to loss of GRHL2 by nascent RNA Bru-seq using an MCF7 conditional knockout model. Integration of ChIP- and Bru-seq pinpoints candidate direct GRHL2 target genes in luminal breast cancer. Multiple connections between GRHL2 and proliferation are uncovered, including transcriptional activation of ETS and E2F transcription factors. Among EMT-related genes, direct regulation of CLDN4 is corroborated but several targets identified in other cells (including *CDH1* and *ZEB1*) are ruled out by both ChIP- and Bru-seq as being directly controlled by GRHL2 in luminal breast cancer cells. Gene clusters correlating positively (including known GRHL2 targets such as ErbB3, CLDN4/7) or negatively (including TGFB1 and TGFBR2) with GRHL2 in the MCF7 knockout model, display similar correlation with GRHL2 in ER positive as well as ER negative breast cancer patients. Altogether, this study uncovers gene sets regulated directly or indirectly by GRHL2 in luminal breast cancer, identifies novel GRHL2-regulated genes, and points to distinct GRHL2 regulation of EMT in luminal breast cancer cells.

**Video Abstract**

**Supplementary Information:**

The online version contains supplementary material available at 10.1186/s12964-022-01029-5.

## Background

The *Grh* gene was discovered in *Drosophila* and its mammalian homologs have three members (*GRHL1, GRHL2 and GRHL3*) [1]. Mice lacking GRHL1, 2, or 3 display neural tube closure defects and a variety of defects in epithelia of several organs with disruption of epithelial adhesion complexes as a major common event [2–7]. GRHLs support expression of genes encoding key epithelial cell–cell junction proteins in desmosomes, adherens junctions, and tight junctions as well as targets involved in cytoskeletal regulation, membrane trafficking, and guidance cues. Several of these genes have been identified as direct GRHL transcriptional targets [3, 4, 8–15]. ChIP-seq in placenta, kidney, and lung epithelial cells has revealed > 5000 GRHL2 binding peaks [11, 14, 15]. GRHL2 depletion in these same tissues identified a few hundred to a thousand genes whose expression was altered. Notably, (i) overlap between these different tissues with respect to GRHL2 binding peaks and candidate target genes is limited pointing to common and tissue specific functions of GRHL2 and (ii) for many of the GRHL2 target genes regulation appears indirect, which may involve GRHL2-regulated expression of other transcription factors or epigenetic modifiers [16, 17].

*GRHL2* is located on chromosome 8q22 that is frequently amplified in many cancers, including breast cancer, colorectal cancer and oral squamous cell carcinoma [18–20]. GRHL2 acts as an activator or suppressor of target gene transcription by interacting with promotor and enhancer regions in competition or in cooperation with other transcription factors and epigenetic regulators [2]. GRHL2 may enhance proliferation, replicative potential, and evasion of cell death through activation of the ErbB3 gene, epigenetically promoting expression of hTERT, and suppressing death receptor expression [9, 18, 19]. Indeed, GRHL2 expression was negatively correlated with metastasis-free survival in breast cancer patients [21, 22]. By contrast, others have reported that high GRHL2 expression in breast cancer cell lines is associated with sensitivity to anoikis and chemotherapy and reduced tumor initiation capacity [23, 24].

Loss of GRHL2 was reported in gastric cancer and GRHL2 was found downregulated at the invasive front of breast cancers and loss of GRHL2 expression in primary breast cancers correlated with lymph node metastasis [9, 25]. A key mechanism by which GRHL2 may suppress aspects of tumor progression is through inhibition of epithelial-to-mesenchymal transition (EMT). GRHL2 acts in a double negative feedback loop with ZEB1 and it activates the expression of miR-200s that, in turn, are in a double negative feedback loop with ZEBs, thereby enforcing the epithelial phenotype [9, 17, 23, 24, 26, 27]. The roles of GRHL2 may be tumor type- and stage-specific through regulating different target genes in different cancers [28].

Breast cancer represents a heterogeneous disease with multiple clinically relevant subtypes appearing to originate from luminal or basal epithelial cells in the duct [29–31]. The luminal subtype accounts for the majority of breast cancer cases and can be treated by therapies targeting estrogen receptor alpha (ER⍺) signaling [32]. Recent studies have shown that GRHL2 cooperates with androgen receptor in prostate cancer [33] and with ER⍺ in breast cancer. Like FOXA1, GRHL2 may act as a pioneer factor, promoting chromatin accessibility and GRHL2 has been found to co-occupy enhancer elements with FOXA1, GATA3, and ER⍺ to regulate ER⍺ signaling output in hormone receptor positive breast cancer [34–37].

In this study, we identify genomic binding sites of GRHL2 shared among 3 luminal breast cancer cell lines and find that only a small subset of these GRHL2 peaks is associated with ER binding sites. We integrate this ChIP-seq data with Bru-seq analysis of genes showing transcriptional responses at different time points after conditional GRHL2 knockout in MCF7 cells. For genes showing sustained up- or downregulation in response to GRHL2 deletion, we explore correlations with GRHL2 expression in breast cancer patients. Our findings reveal gene sets regulated directly or indirectly by GRHL2 in luminal breast cancer that partly overlap but also appear markedly distinct from targets identified in other tissues.

## Methods

### Cell lines and plasmids

Human breast cancer cell lines representing the luminal subtype (MCF7, T47D and BT474) were obtained from the American Type Culture Collection. The Hs578T human basal-B breast cancer cell line served as a GRHL2-negative control. Cells were cultured in RPMI1640 medium with 10% fetal bovine serum, 25 U/mL penicillin and 25 µg/mL streptomycin in the incubator (37 °C, 5% CO_2_). For production of lentiviral particles, VSV, GAG, REV and Cas9 or single guide (sg) RNA plasmids were transfected into HEK293 cells using Polyethylenimine (PEI). After 2 days, lentiviral particles were harvested and filtered. Conditional Cas9 cells were generated by infecting parental cells with lentiviral particles expressing the Edit-R Tre3G promotor-driven Cas9 (Dharmacon) and selected by blasticidin. Limited dilution was used to generate Cas9 monoclonal cells. Subsequently, Cas9-monoclonal cells were transduced with U6-gRNA:hPGK-puro-2A-tBFP control non-targeting sgRNAs or GRHL2-specific sgRNAs (Sigma) and selected by puromycin. The EHF plasmid was kindly provided by Dr. Giuseppina Carbone, Institute of Oncology Research, Bellinzona, Switzerland and described previously [38, 39]. The EHF plasmid was transfected into cells using Lipofectamin 2000 according to a protocol provided by the manufacturer.

### Western blot

Cells were lysed by radioimmunoprecipitation (RIPA) buffer (150 mM NaCl, 1% Triton X-100, 0.5% sodium deoxycholate and 0.1% Tris and 1% protease cocktail inhibitor (Sigma-Aldrich. P8340)). Lysates were sonicated and protein concentration was determined by bicinchoninic acid (BCA) assay. Cell lysates were mixed with protein loading buffer, separated by SDS-PAGE, and transferred to a methanol-activated polyvinylidene difluoride (PVDF) membrane (Milipore, The Netherlands). The membrane was blocked with 5% bovine serum albumin (BSA; Sigma-Aldrich) for 1 h at room temperature (RT). Next, membranes were stained with primary antibody overnight at 4 °C and HRP-conjugated secondary antibodies for half hour at room temperature (RT). After staining with Prime ECL Detection Reagent (GE Healthcare Life science), chemoluminescence was detected with an Amersham Imager 600 (GE Healthcare Life science, The Netherlands). The following antibodies were used: GRHL2 (Atlas-Antibodies, hpa004820) Cas9 (Cell Signaling, 14,697), and GAPDH (SantaCruz, sc-32233).

### ChIP-seq

Cells were grown in RPMI-1640 complete, serum-containing medium. Cross-linking was performed by 1% formaldehyde for 10 min at room temperature (RT). Then 1 M glycine (141 µl of 1 M glycine for 1 ml of medium) was used to quench for 5 min at RT. Cells were washed twice with ice-cold PBS containing 5 µl/ml phenylmethylsulfonyl fluoride (PMSF). Cells were harvested by centrifugation (2095 g for 5 min at 4 °C) and lysed with NP40 buffer (150 mM NaCl, 50 mM Tris–HCl, 5 mM EDTA, 0.5% NP40, 1% Triton X-100) containing 0.1% SDS, 0.5% sodium deoxycholate and protease inhibitor cocktail (EDTA-free Protease Inhibitor Cocktail, Sigma). Chromatin was sonicated to an average size of 300 bp (Additional file 1: Fig. S1). GRHL2-bound chromatin fragments were immunoprecipitated with anti-GRHL2 antibody (Sigma; HPA004820). Precipitates were washed by NP buffer, low salt (0.1% SDS, 1% Triton X-100, 2 mM EDTA, 20 mM Tris–HCl (pH 8.1), 150 mM NaCl), high salt (0.1% SDS, 1% Triton X-100, 2 mM EDTA, 20 mM Tris–HCl (pH 8.1), 500 mM NaCl) and LiCl buffer (0.25 M LiCl, 1%NP40, 1% deoxycholate, 1 mM EDTA, 10 mM Tris–HCl (pH 8.1)). Chromatin was de-crosslinked by 1% SDS at 65 °C. DNA was purified by Phenol:Chloroform:Isoamyl Alcohol (PCI) and then diluted in TE buffer.

In order to examine the quality of our samples before sequencing, ChIP-qPCR (quantitative polymerase chain reaction) was performed to validate interaction of GRHL2 with the promoter region of Claudin-4 (*CLDN4*), a known direct target gene of GRHL2 [4]. The results confirmed the GRHL2 binding site around the *CLDN4* promoter (Additional file 2: Fig. S2). The following primers were used for ChIP-qPCR: *CLDN4* forward: gtgacctcagcatgggctttga, *CLDN4* reverse: ctcctcctgaccagtttctctg, Control (an intergenic region upstream of the *GAPDH* locus) forward: atgggtgccactggggatct, Control reverse: tgccaaagcctaggggaaga, *ZEB1* promoter^#^ forward: cggtccctagcaacaaggtt, *ZEB1* promoter^#^ reverse: tcgcttgtgtctaaatgctcg. *ZEB1*^##^ forward: gccgccgagcctccaacttt, *ZEB1*^##^ reverse: tgctagggaccgggcggttt, *OVOL2* exon forward: ccttaaatcgcgagtgagacc, *OVOL2* exon reverse: gtagcgagcttgttgacacc, *CDH1* intron forward: gtatgaacggcaagcctctg, *CDH1* intron reverse: caagggagccaggaagagaa. ChIP-qPCR data were collected and analyzed using the 2^−ΔΔCt^ method [40].

For ChIP-seq, library preparation and paired-end (151 bp) sequencing were performed by GenomeScan (Leiden, The Netherlands). MCF7, T47D and BT474 had 87,393,758, 84,633,440, and 82,080,866 pair-end reads, respectively.

### ChIP-seq analysis

Less than 5% of adapter sequences were present, and the mean per base sequence quality was > 30, indicating high quality reads and no requirement for adapter-trimming (Additional files 3 and 4: Figs. S3 and S4). Paired-end reads were mapped to the human reference genome (hg38) using BWA-MEM [41] with default parameters. Over 93% of total reads were mapped to the human genome in T47D and MCF7 and 57.3% in BT474. Phred quality score (Q score) was used to measure base calling accuracy [42]. Q > 30 scores (corresponding to a 0.1% error rate [43]) were > 86% in T47D and MCF7 and 48.6% in BT474. Reads with low mapping quality (≤ Q30) were filtered out. MACS version 2.1.0 [44] was used for peak calling by default settings. The q value was adjusted to 0.1 for BT474 cell line to avoid loss of peaks. The annotatePeaks and MergePeaks functions from HOMER [45] were used to annotate and overlap peaks, respectively. ChIPseeker was used for the analysis of ChIP-seq peaks coverage plot and the density profile of GRHL2 binding sites [46]. Motif analysis was performed using ChIP-seq peaks with high scores by the MEME-ChIP program with default settings. ChIP-seq data was visualized by the UCSC genome browser. To analyze coverage of GRHL2 peaks at consensus motifs for GRHL2, ER⍺, FOXA1, and GATA3 binding, the JASPAR 2022 database was used to identify motifs [47]. To analyze colocalization of our GRHL2 binding events with published ER⍺ peaks in luminal breast cancer cells, ChIP-seq data files from a study mapping ER⍺ binding sites in MCF7, BT474, and T47D [48] were intersected using bedtools (v2.3.0) [PMID: 20110278] and ChIP-seq data files from two different studies mapping ER-alpha binding sites in MCF7 were intersected [49, 50].

### Bru-seq

MCF7 cells expressing inducible Cas9 and control non-targeting sgRNAs or GRHL2-specific sgRNAs were exposed to 1 µg/ml doxycycline. At different timepoints after doxycycline-induced deletion of GRHL2, cells were incubated with a final concentration of 2 mM Bru at 37 °C for 30 min. Cells were lysed in TRIzol reagent (Sigma) and Bru-labelled nascent RNA was isolated using an anti-BrdU antibody conjugated to magnetic beads [51]. Subsequently, cDNA libraries were generated using the Illumina TruSeq library kit and sequenced using the Illumina NovaSeq 6000 Sequencing System. Sequence reads were strand-specific, paired-ended with read lengths of ~ 150 nucleotides. Reads were pre-mapped to the ribosomal RNA (rRNA) repeating unit (GenBank U13369.1) and the mitochondrial and EBV genomes (from the hg38 analysis set) using Bowtie2 (2.3.3). Unaligned reads were subsequently mapped to human genome build hg38/GRCh38 using STAR (v 2.5.3a) and a STAR index created from GENCODE annotation version 27 [51, 52].

### Bru-seq analysis

To identify GRHL2-regulated genes, an inter-sample comparison analysis was performed comparing RPKM (reads per kilobase per million mapped reads) for each gene in the doxycycline-treated samples compared to the untreated sample, to obtain fold-change (FC) and *p* values. Genes with *p* < 0.05 and FC > 2 or FC < 0.5 in any of the doxycycline-treated samples relative to untreated cells were filtered. Subsequently, genes responding to Cas9 induction in the context of both GRHL2 sgRNAs were selected and genes responding also in the context of control sgRNA were eliminated from this list. A heatmap was generated by R. The function “fviz_nbclust()” from the R package “factorextra” was used to determine and visualize the optimal numbers of clusters using the method “within cluster sums of square”. The STRING database (version 11.5) was used to assign protein interaction networks to Bru-seq data [53].

### Breast cancer patient mRNA expression data analysis

A compendium microarray dataset, all Affymetrix U133a, was used, containing RNA expression data of primary tumors of 867 untreated, lymph node negative patients (MA-867 dataset [59]; publicly available at GSE2034, GSE5327, GSE2990, GSE7390 and GSE11121). Raw.cel files were downloaded, processed with fRMA and batch effects were corrected using ComBat.

RNAseq data retrieved from the Molecular Taxonomy of Breast Cancer International Consortium (METABRIC) data set [54, 55] was used consisting of targeted sequencing data of 1904 primary breast tumors with matched normal tissues. Data visualization and calculation of co-expression z-scores were performed using cBioPortal (https://www.cbioportal.org/).

### SRB assay

For Sulforhodamine B (SRB) assays, cells were seeded into 96-well plates. At indicated time points, cells were fixed with 50% Trichloroacetic acid (TCA, Sigma-Aldrich) for 1 h at 4 °C and then plates were washed with demineralized water four times and air-dried at RT. Subsequently, 0.4% SRB (60 µl/well) was added and kept for at least 2 h at RT. The plates were washed five times with 1% acetic acid and air-dried. 10 mM (150 µl/well) Tris was added and kept for half hour at RT with gentle shaking. The absorbance value was measured by a plate-reader Fluostar OPTIMA.

## Results

### Genome-wide identification of GRHL2 binding sites in luminal breast cancer cells

To identify GRHL2 binding sites, ChIP-seq was performed in the human luminal breast cancer cell lines, MCF7, T47D and BT474. As a quality control of the ChIP samples, ChIP-qPCR confirmed the interaction of GRHL2 with the promoter region of *CLDN4*, a known direct target gene of GRHL2 [4] in all three luminal, GRHL2-positive cell lines but not in the GRHL2-negative Hs578T human basal-B breast cancer cell line (Additional file 2: Fig. S2). Subsequently, ChIP-seq was performed and the coverage of peak regions across chromosomes was analyzed [46]. In each sample, GRHL2 was associated with all chromosomes (Additional file 5: Fig. S5).

**Fig. 1:**
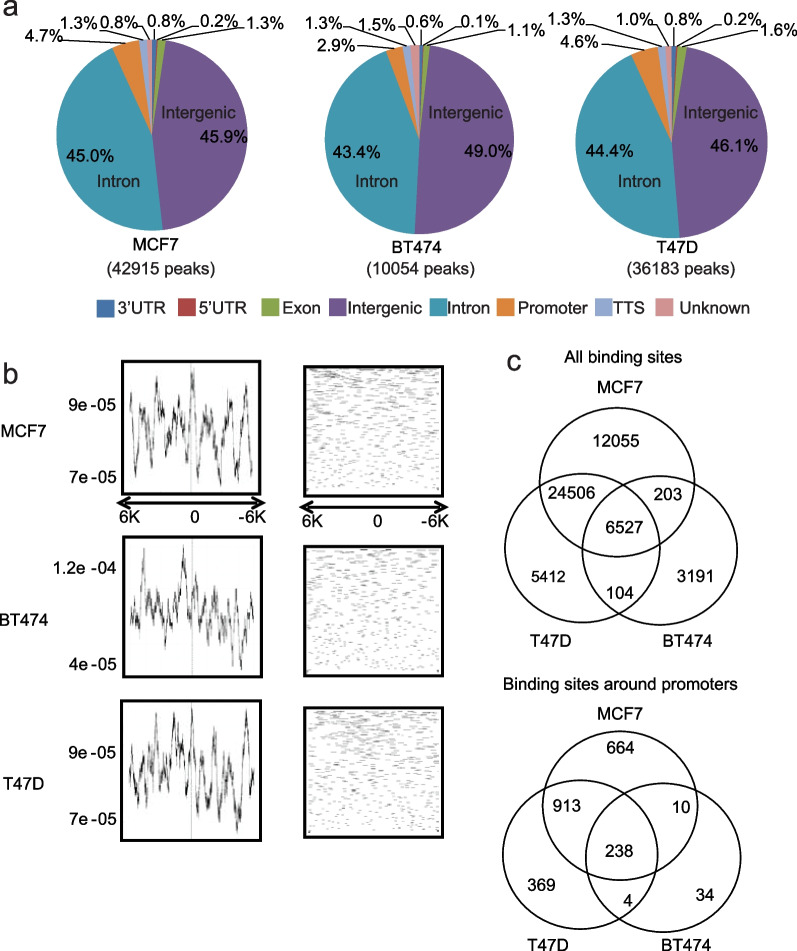
GRHL2 ChIP-seq in luminal breast cancer cells. **a** Percentage of GRHL2 binding sites found at promoter regions, 5′ untranslated regions (UTRs), 3′ UTRs, exons, introns, intergenic regions, transcription termination sites (TTSs) and unknown regions in the indicated luminal breast cancer cell lines. Promoter regions are defined as − 1000 to + 100 bp from the transcription start sites (TSS). **b** Read count frequency and density profile of GRHL2 binding sites within − 6000 to + 6000 bp of the TSS. Left panels show GRHL2 ChIP-seq read count frequencies in indicated cell lines (Y axis, read count frequency; X axis, genomic region). Right panels show density of ChIP-seq reads for GRHL2 binding sites in the indicated cell lines. **c** Venn diagrams showing overlap of GRHL2 binding sites among the three indicated cell lines. Top panel shows overlap for all peaks. Bottom panel shows overlap for peaks within the − 1000 to + 100 promoter region

GRHL2 binding sites were mainly located in intergenic regions and introns, with ~ 3–5% of the peaks located in − 1000 to + 100 bp promoter regions (Fig. 1a). Analysis of read count frequency and density profiling of GRHL2 binding sites within − 6000 to + 6000 bp of the transcription start site (TSS) showed no enrichment around the TSS (Fig. 1b). Intersection of the data in the 3 cell lines identified 6527 conserved GRHL2 binding sites in luminal breast cancer cells. Of these, 238 binding sites located in the − 1000 to + 100 bp regions, representing candidate interactions for direct GRHL2-mediated regulation of gene promoter activity (Fig. 1c; Additional file 6: Table S1).

### A small proportion of GRHL2 peaks is associated with ER⍺ binding

**Fig. 2:**
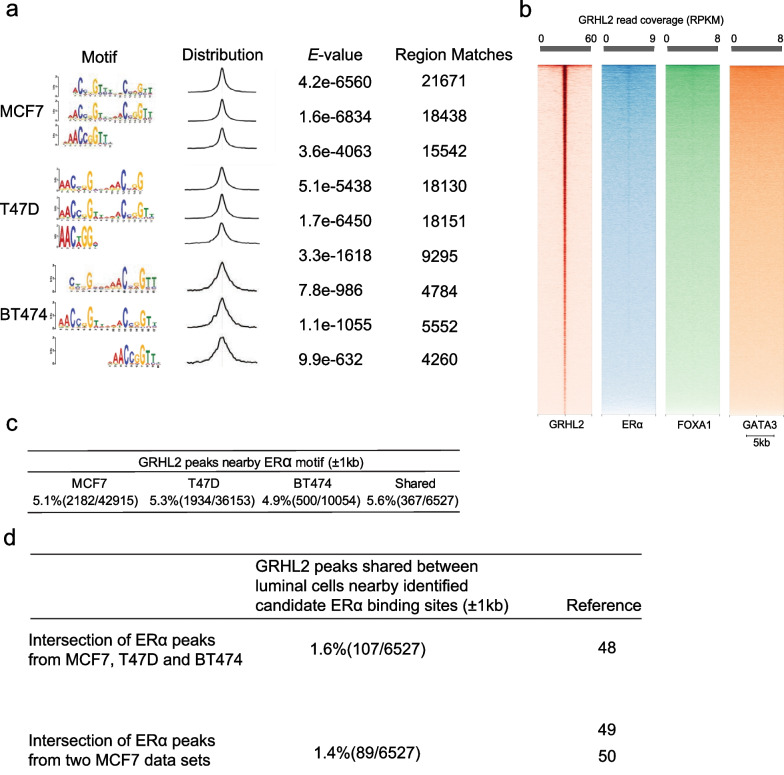
Association of GRHL2 motif with ER transcriptional complex in luminal breast cancer cells. **a** DNA-binding motif of GRHL2 in luminal breast cancer. From left to right, the first panel shows the identified motifs in the indicated cell lines. The second panel shows distribution of the best matches to the motif in the sequences. The third panel shows the *E*-value, representing the significance of the motif according to the motif discovery. The last panel shows the number of regions that match the corresponding motif. **b** Heatmaps showing the coverage of identified GRHL2 peaks shared between MCF7, BT474 and T47D at GRHL2 motifs (red) (n = 20,766), ER⍺ motifs (blue) (n = 76,564), FOXA1 motifs (Green) (n = 88,923) and GATA3 motifs (Orange) (n = 93,403). Note that the read coverage scale differs for the different heatmaps. **c** Table indicating the occurrence of ER⍺ consensus motif in a region spanning 1000 bp up- and downstream of all GRHL2 peaks either identified in the indicated cell lines (left 3 columns) or shared between the indicated cell lines (right column). **d** Table indicating the occurrence of published ER⍺ binding events in a region spanning 1000 bp up- and downstream of all GRHL2 peaks shared between MCF7, BT474 and T47D (upper row) or shared between 2 MCF7 datasets (bottom row)

MEME-ChIP identified 3 GRHL2 binding motifs with low *E* values in each cell line (Fig. 2a), whose core binding site matched previously published motifs [14, 15, 17, 56]. Based on the published interaction of GRHL2 with ER⍺, FOXA1, and GATA3 at enhancer elements of target genes [34–36, 57], we addressed to what extent the identified conserved GRHL2 binding sites in luminal breast cancer cells were flanked by putative binding sites for the ER⍺-mediated transcriptional complex. Heatmap visualization showed concentration of the GRHL2 peaks at the consensus GRHL2 motif [AACCGGTT] as expected (Fig. 2b). GRHL2 peaks showed only a weak trace for ER⍺ [AGGTCAnnnTGACCT] and a barely detectable trace for the FOXA1 motif [TGTTT(A/G)C], and no concentration of the GATA3-binding motif [A/T)GATA(A/G] was observed. Indeed, among the shared GRHL2 peaks in luminal breast cancer cells ~ 5% was flanked by an ER⍺ binding motif within ± 1000 bp (Fig. 2c).

To further address colocalization of GRHL2 and ER⍺ binding in luminal breast cancer, regions flanking ± 1000 bp of the conserved GRHL2 peaks in MCF7, BT474, and T47D were interrogated for the presence of previously reported ER⍺ binding events. For this purpose, ChIP-seq data files from a study mapping ER⍺ binding sites in MCF7, BT474, and T47D [48] and ChIP-seq data files from two studies mapping ER⍺ binding sites in MCF7 were intersected [49, 50]. These studies had used similar culture conditions as ours, using phenol red medium and serum containing estrogen. Only a minor fraction of ~ 1.5% of conserved GRHL2 peaks identified in our study was flanked by established ER⍺ binding sites in luminal breast cancer cells identified in those studies (Fig. 2d). Altogether, this data indicated that the majority of GRHL2 binding sites in luminal breast cancer cells were not associated with the ER⍺-mediated transcriptional complex.

### Changes in gene transcription in response to GRHL2 loss

**Fig. 3:**
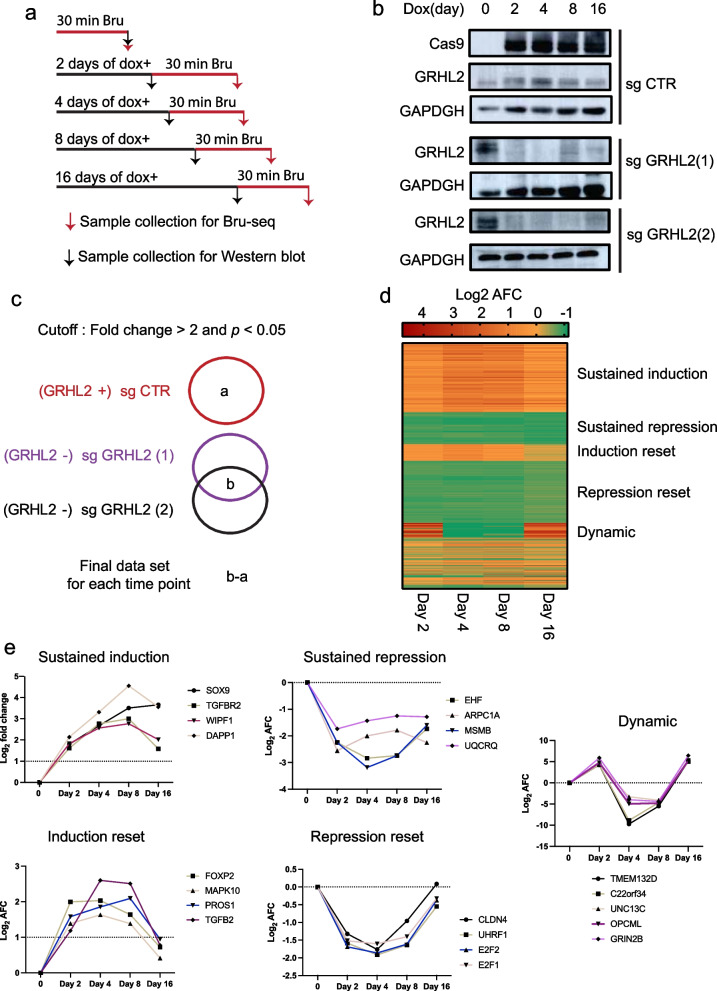
Bru-seq analysis of transcriptional changes in response to GRHL2 loss in luminal breast cancer MCF7 cells. **a** Bru-seq sample preparation**.** Bromouridine (Bru) labeling of nascent RNA was carried out for 30 min at the indicated time points after doxycycline (dox)-induced GRHL2 deletion. **b** Western blot analysis of GRHL2 expression levels at the indicated time points in sgCTR and sgGRHL2 transduced MCF7 cells. Cas9 induction is monitored and GAPDH serves as loading control. **c** Bru-seq data analysis approach. Each circle represents a gene set with differential transcription relative to the condition where no doxycycline was added. **d** Heatmap for genes whose transcription was altered in response to GRHL2 depletion. **e** Graphs depicting clusters of genes with distinct patterns of transcriptional changes in response to GRHL2 depletion. Graphs represent log_2_ AFC of transcription in sgGRHL2(1) and sgGRHL2(2) cells. “Dynamic”: genes with AFC > 2; *p* < 0.05 at some and AFC < 0.5; *p* < 0.05 at other time points. “Sustained induction”: genes with AFC > 2; *p* < 0.05 at all time points. “Sustained repression”: genes with AFC < 0.5; *p* < 0.05 at all time points. “Induction reset”: genes with AFC > 2; *p* < 0.05 at early time points followed by a return to 1 < AFC < 2 at day 16. “Repression reset”: genes with AFC < 0.5; *p* < 0.05 at early time points followed by a return to 0.5 < AFC < 1 at day 16

Next, we employed nascent RNA Bru-seq to investigate genome-wide dynamic changes in DNA transcription triggered by GRHL2 loss. For this purpose, we made use of a conditional Cas9 MCF7 knockout model expressing a control or 2 different GRHL2 sgRNAs (sgCTR, sgGRHL2(1) and sgGRHL2(2), respectively). At 0, 2, 4, 8, or 16 days after GRHL2 knockout, cells were incubated with bromouridine (BrU) for 30 min to label nascent RNA (Fig. 3a) and analyzed in parallel by Western blot for the induction of Cas9 and deletion of GRHL2 (Fig. 3b; Additional file 7: Fig.S6). To identify GRHL2-regulated genes, for each time point, the average fold change (AFC) of transcription induced by doxycycline treatment in the two sgGRHL2 and sgCTR samples was determined. 262 genes were found to be upregulated and 226 genes were downregulated in at least one time point after GRHL2 loss in both sgGRHL2 samples (FC > 2 or FC < 0.5; *p* < 0.05) but not in the sgCTR samples (Fig. 3c; Additional file 8: Table S2).

GRHL2-regulated genes were clustered in a heatmap using the AFC at each time point (Fig. 3d). Five clusters were identified based on transcriptional dynamics (Fig. 3e; Additional file 8: Table S2). There was no preference for the subset of genes containing GRHL2 binding sites flanked by ER⍺ binding in either of the clusters. Clusters displaying sustained upregulation of RNA synthesis or a transient induction that subsequently returned to baseline included *TGFB1, TGFB2,* and *TGFBR2* pointing to enhanced TGFß signaling. Other clusters showed sustained downregulation of RNA synthesis following GRHL2 deletion or a transient repression that subsequently returned to baseline. These included genes encoding the epithelial specific ETS transcription factor EHF, the *E2F1* and *E2F2* genes encoding E2F transcription factors involved in cell cycle progression, and the *CLDN4* gene encoding an epithelial tight junction protein. Another cluster showed responses that could be categorized as highly dynamic with alternating increased and decreased transcription.

### Identification of candidate genes regulated by GRHL2 promoter binding

GRHL2 can regulate gene transcription through interaction with gene promoter or enhancer elements [2]. We intersected the list of genes whose expression levels were significantly altered after GRHL2 loss in MCF7 at one or more time points as identified by Bru-seq, with genes harboring GRHL2 binding sites in the − 1000 to + 100 bp promoter regions in MCF7 identified by ChIP-seq. 53 genes were identified where transcriptional regulation could be explained by direct GRHL2 interactions at the promotor region (Additional file 8: Table S2; genes indicated in bold). Restricting this list to genes harboring GRHL2 binding sites in the promoter regions that were shared in all three luminal breast cancer cell lines, reduced this number to 9 (Additional file 6: Table S1; genes indicated in bold). The presence or absence of GRHL2 binding sites in the promoter region did not correspond to the dynamic pattern of the transcriptional response of the gene (Additional file 8: Table S2). Together, this indicated that the majority of the genes showing a transcriptional response to GRHL2 depletion was regulated either by direct interactions at enhancer elements or indirectly, e.g., through GRHL2 regulation of a transcription factor targeting the gene of interest.

### EHF is a direct GRHL2-target inversely correlated with GRHL2 in breast cancer subtypes

**Fig. 4:**
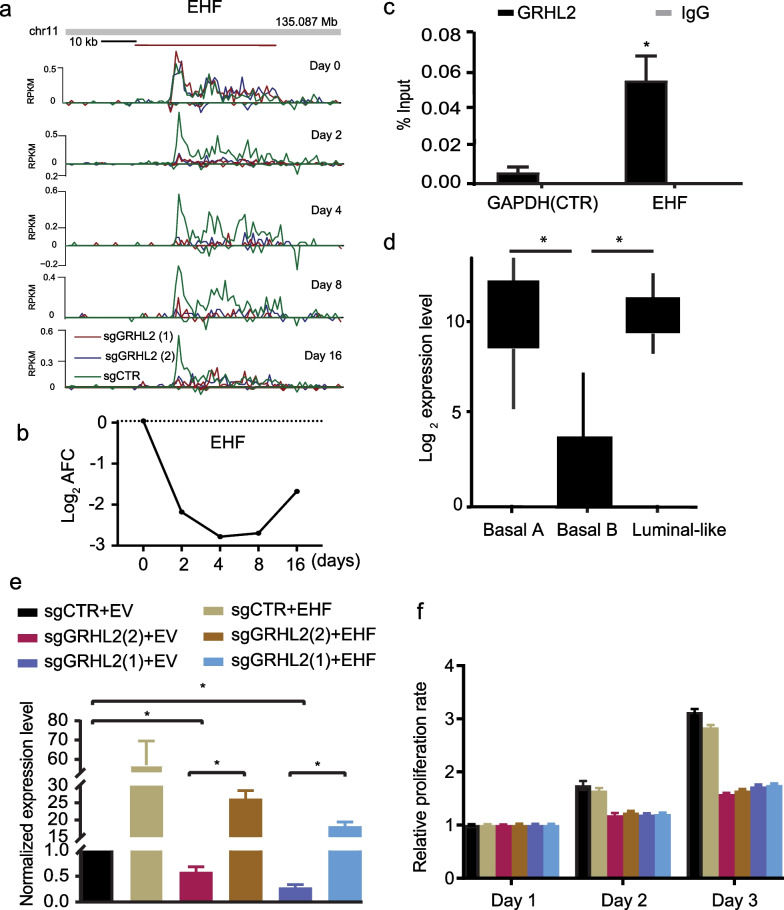
EHF represents a direct GRHL2 regulated gene. **a** Bru-seq reads for *EHF* at indicated time points after to GRHL2 deletion. Track colors: green, sgCTR; red, sgGRHL2(1); blue, sgGRHL2(2). Positive y-axis indicates the plus-strand signal of RNA synthesis from left to right and the negative y-axis represents the minus-strand signal of RNA synthesis from right to left. **b** Line graph depicting the log_2_ AFC of *EHF* transcription in sgGRHL2(1) and sgGRHL2(2) cells. **c** ChIP-qPCR showing enrichment of GRHL2 binding sites in *EHF* promoter region but not in the control *GAPDH* gene. Graph represents the efficiency of indicated genomic DNA co-precipitation with anti-GRHL2 Ab (black bars) or IgG control Ab (grey bars). Signals for IgG control and GRHL2 antibody pulldown samples are normalized to input DNA and are presented as % input with SEM from 3 technical replicates. Data are statistically analyzed by t-test and *indicates *p* < 0.05. **d** *EHF* mRNA expression in a panel of 52 human breast cancer cell lines covering luminal-, basal A-, and basal B subtypes extracted from RNA-seq data. Data is statistically analyzed by t-test and *indicates *p* < 0.05. **e** qRT-PCR analysis of expression level of *EHF* mRNA after 4 days of doxycycline treatment of MCF7 cells transduced with dox-inducible Cas9 and sgCTR or sgGRHL2 constructs, in combination with ectopic expression of *EHF* or empty vector (EV) plasmids. Data are presented as mean ± SEM from three technical replicates. Data are statistically analyzed by t-test. *Indicates *p* < 0.05. **f** Graph showing results from SRB assay after 4 days doxycycline-treatment as in (**e**) and subsequent culture for the indicated time periods

EHF was identified as a GRHL2 target harboring a GRHL2 binding site in its promoter region that was conserved in all three luminal breast cancer cell lines (Fig. 3e; Additional files 6 and 8: Tables S1 and S2). EHF had not been previously reported as a GRHL2 target gene while our Bru-seq tracks showed that EHF transcription was rapidly and continuously attenuated following GRHL2 loss (Fig. 4a, b). ChIP-qPCR confirmed the interaction between GRHL2 and the promoter region of the *EHF* gene (Fig. 4c). *EHF* is a member of the ETS transcription factor subfamily characterized by epithelial-specific expression [58]. Epithelial markers (e.g., GRHL2, CLDN4 and E-cadherin) are lost in basal B breast cancer cells as compared to the luminal and basal A subtype and we examined whether *EHF* expression followed this pattern. Indeed, RNA-seq data from a panel of 52 human breast cancer cell lines [59] showed a decrease of *EHF* RNA levels in the basal B subtype (Fig. 4d).

**Fig. 5:**
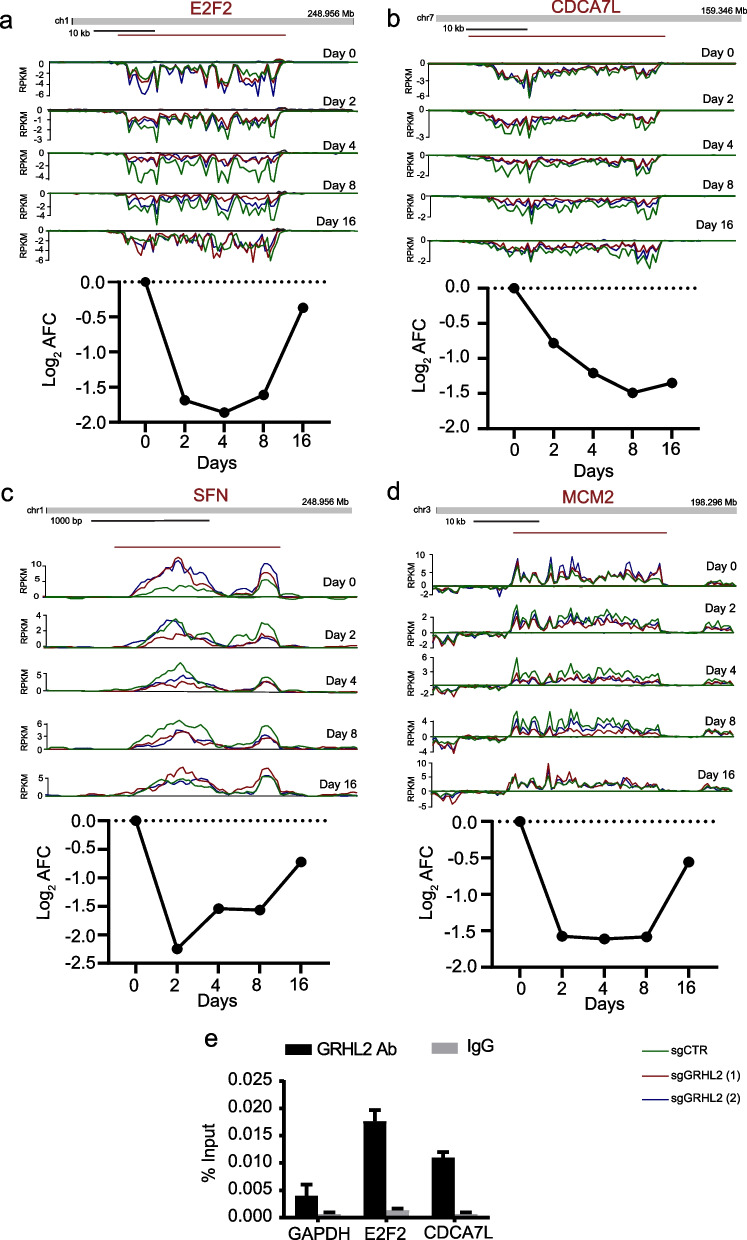
Downregulation of RNA synthesis for genes involved in cell cycle progression after GRHL2 loss. **a**–**d** Top: Bru-seq reads for indicated genes at indicated time point after to GRHL2 deletion. Track colors: green, sgCTR; red, sgGRHL2(1); blue, sgGRHL2(2). Bottom: Line graphs depicting the log_2_ AFC of transcription in sgGRHL2(1) and sgGRHL2(2) cells for the indicated genes. The positive y-axis indicates the plus-strand signal of RNA synthesis from left to right and the negative y-axis represents the minus-strand signal of RNA synthesis from right to left. **e** Validation of interaction of GRHL2 binding sites with the promoter regions of indicated genes by ChIP-qPCR**.** Signals for IgG control and GRHL2 antibody pulldown samples are normalized to input DNA and are presented as % input with SEM from 3 technical replicates. Data are statistically analyzed by t-test and *indicates *p* < 0.05

Studies in various cancer types have attributed tumor promoting as well as tumor suppressive roles to EHF but its role in breast cancer is largely unknown [60]. GRHL2 loss led to a rapid reduction in MCF7 cell growth and we tested whether ectopically overexpressed *EHF* could enhance proliferation in absence of GRHL2. However, overexpression of *EHF* did not rescue cell proliferation of GRHL2 KO MCF7 cells (Fig. 4e, f). The RNA synthesis rates of several other genes supporting cell cycle progression were rapidly suppressed in response to GRHL2 loss, including E2F transcription factors *E2F1* and *E2F2* and other genes such as *CDCA7L* and *MCM2* [61–63] (Figs. 5a–d, 7a). Our ChIP-seq data revealed GRHL2 binding sites in the promoter regions of *E2F2* and *CDCA7L* in MCF7 (Additional file 8: Table S2) and this finding was corroborated by ChIP-qPCR analysis (Fig. 5e). Altogether, these results showed that several genes involved in cell cycle progression are rapidly downregulated following GRHL2 depletion with *EHF, E2F2,* and *CDCA7L* representing candidate targets for direct transcriptional regulation by GRHL2 at the gene promoter.

### Regulation of EMT-related genes: CLDN4 but not CDH1, ZEB1, and ZEB2 represent direct GRHL2 targets in luminal breast cancer

GRHL2 and OVOL2 support an epithelial phenotype and counteract EMT transcription factors such as ZEB1, ZEB2, and SNAIL. Genes encoding epithelial adhesion components such as CLDN4 in tight junctions or E-cadherin (CDH1) in adherens junctions are regulated by this balance [64]. It has been reported that GRHL2 binding sites are present in the intronic region of *CDH1* and in the promoter regions of *CLDN4* and *OVOL2* for activation of transcription, and GRHL2 was reported to bind the *ZEB1* gene as a negative regulator [4, 12, 15, 23, 24, 65].

**Fig. 6:**
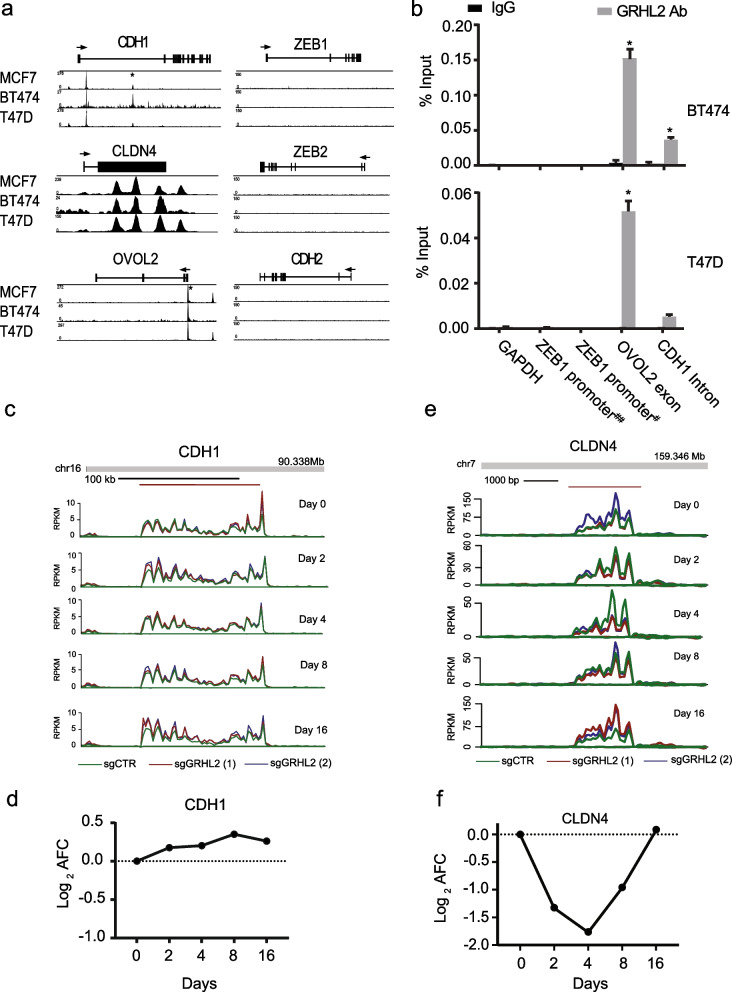
Regulation of EMT related genes by GRHL2. **a** ChIP tracks for the indicated genes in three luminal breast cancer cell lines. The track height is scaled from 0 to the indicated number. The locus with its exon/intron structure is presented above the tracks. *Indicates binding sites validated by ChIP-qPCR in (b). **b** ChIP-qPCR validation of presence and absence of GRHL2 binding sites identified by ChIP-seq. Graphs represent the efficiency of indicated genomic DNA co-precipitation with anti-GRHL2 Ab (grey bars) or IgG control Ab (black bars). Note enrichment of GRHL2 binding at *OVOL2* exon and *CDH1* intron, but not at *ZEB1* promoter regions. For *ZEB1* detection, ChIP-qPCR was performed using primers that have been previously reported to amplify *ZEB1* promoter DNA sequences bound by GRHL2 in human mammary epithelial cells and in PEO1 but not OVCA429 human ovarian cancer cells (indicated by ##) [17, 23] and another primer set that did not confirm GRHL2 promoter interaction in ovarian cancer cells (indicated by #) [17]. Signals for IgG control and GRHL2 antibody pulldown samples were normalized to input DNA and presented as % input with SEM from 3 technical replicates. Data were statistically analyzed by t-test and * indicates *p* < 0.05. **c**, **e** Bru-seq reads for indicated genes at indicated time point after to GRHL2 deletion. Track colors: green, sgCTR; red, sgGRHL2(1); blue, sgGRHL2(2). **d**, **f** Line graphs depicting the log_2_ AFC of transcription in sgGRHL2(1) and sgGRHL2(2) cells for the indicated genes. The positive y-axis indicates the plus-strand signal of RNA synthesis from left to right and the negative y-axis represents the minus-strand signal of RNA synthesis from right to left

In our ChIP-seq data, a conserved intronic GRHL2 binding site was observed in *CDH1* that was validated by ChIP-qPCR (Fig. 6a, b). However, while GRHL2 was found to transcriptionally activate *CDH1* in earlier reports [4, 21, 33] we did not observe downregulation of *CDH1* nascent RNA synthesis in the first 16 days after GRHL2 loss (Fig. 6c, d). No GRHL2 peaks were associated with *CDH2* (encoding N-cadherin, a mesenchymal marker) while GRHL2 binding was conserved in the promoter regions of *CLDN4* and *OVOL2* (Fig. 6a, b; Additional file 2: Fig S2). *CLDN4* also showed multiple GRHL2 binding sites across the coding and non-coding regions. *CLDN4* transcription was suppressed at 2, 4, and 8 days after GRHL2 depletion but recovered at 16 days (Additional file 8: Table S2; Fig. 6c, d) whereas *OVOL2* was not affected (data not shown).

No GRHL2 binding was observed at the promoter or other regions of *ZEB1* or *ZEB2* as opposed to findings in mammary epithelial cells [24] (Fig. 6a). ChIP-qPCR was performed using primers that have been previously reported to amplify *ZEB1* promoter DNA sequences bound by GRHL2 in human mammary epithelial cells and in PEO1 but not OVCA429 human ovarian cancer cells [17, 23] and another primer set that did not detect GRHL2 promoter interaction in ovarian cancer cells [17] (Fig. 6b). This confirmed the absence of GRHL2 binding in the promoter of *ZEB1* in luminal breast cancer cells. In agreement, no significant changes in transcription of *ZEB1* and *ZEB2* genes were observed after GRHL2 loss in MCF7 (data not shown). Together, these results indicated that *CLDN4* is a direct GRHL2 target while *CDH1, ZEB1, or ZEB2* are unlikely to represent direct GRHL2 target genes in luminal breast cancer cells. These latter genes may be regulated at later timepoints indirectly through other transcriptional regulators [66] or by GRHL2-mediated post-transcriptional modification [17, 23, 67].

### Validation of GRHL2 associations in breast cancer patients

**Fig. 7:**
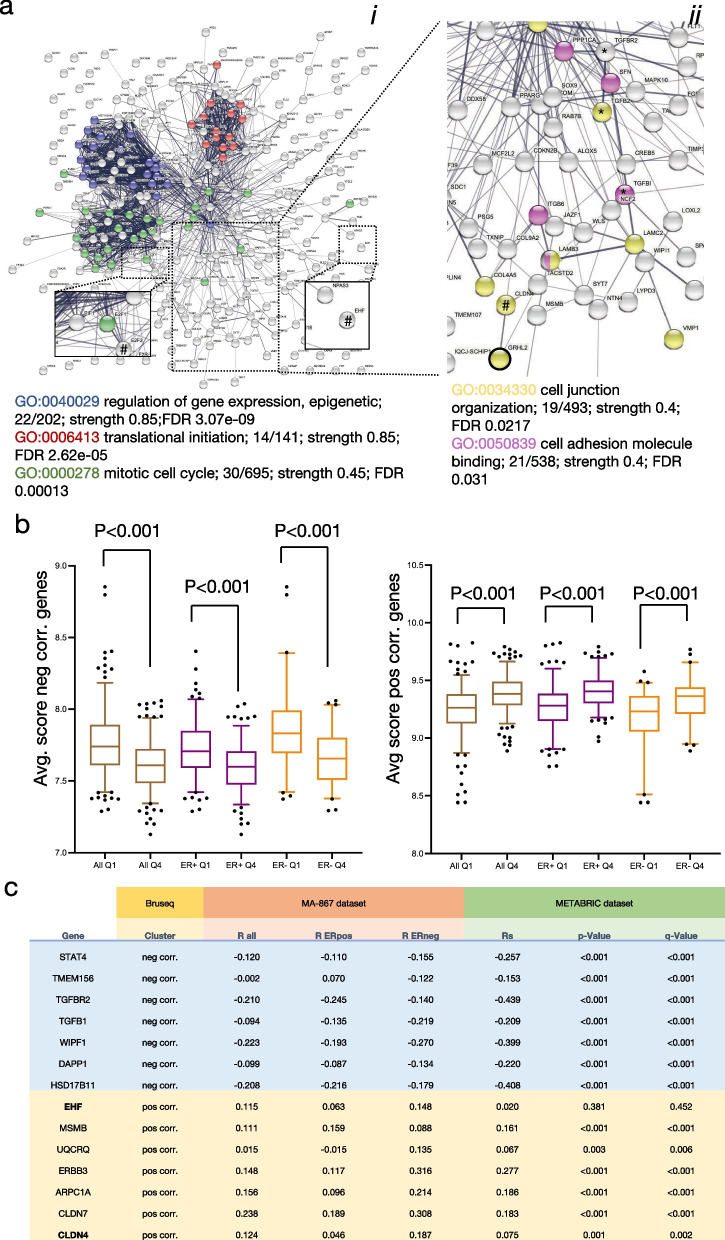
Gene clusters responding to GRHL2 depletion and their correlation with GRHL2 in breast cancer tissues. **a** STRING derived protein interaction analysis of genes displaying sustained up- or down regulation in response to GRHL2 depletion in MCF7. GO terms are color marked as indicated. *i*, entire network with boxes showing zoom-in on indicated regions; *ii*, zoom in on indicated region showing different GO terms. # Indicates GRHL2 targets identified by promoter binding. *Indicates TGFß signaling axis. **b** Average expression (log2 scale) in the MA-867 patient dataset of the cluster of genes negatively (left panel) or positively associated with GRHL2 (right panel) in MCF7 KO model. Patients were divided in 4 quartiles according to the level of GRHL2 expression. Q1, lowest GRHL2 expression; Q4, GRHL2 highest expression; All, all patients grouped together; ER + , ER positive patients grouped; ER-, ER negative patients grouped. Boxplots display the median with 25–75th percentile and dots represent lower 5% and upper 95% samples. *p* values determined by t test (two-sided). **c** Correlation with GRHL2 in MA-867 and METABRIC datasets for indicated genes negatively or positively correlated with GRHL2 in MCF KO model analyzed by Bru-seq. For MA-867 dataset, R-values for all patients grouped together, ER positive patients, or ER negative patients are shown. For METABRIC dataset, correlation, *p*-value, and *q*-values are shown as determined in BioPortal

All genes identified by Bru-seq in the MCF7 conditional GRHL2 KO model falling in the categories “sustained induction/repression” or “induction/repression reset”, were imported in the STRING database to visualize clusters representing enriched functionalities regulated by GRHL2. Three clusters of proteins associated with (i) epigenetic regulation of gene expression (including proteins also connected to GO:0,098,532, histone H3-K27 trimethylation; not shown), (ii) translation initiation, and (iii) mitosis were clearly visible (Fig. 7a). This was in agreement with the growth suppression observed in response to GRHL2 depletion (Fig. 4) and earlier reports involving GRHL2 in histone methylation [17]. E2F1 and E2F2 were connected to the mitosis cluster but EHF showed no connections. No connections of these clusters with GRHL2 were visible but the interaction of GRHL2 with CLDN4 was shown as well as co-expression of GRHL2 with TACSTD2, a transmembrane receptor regulating cell proliferation and migration in development and cancer [68]. The TGFB1, TGFB2, and TGFBR2 axis was not closely connected to GRHL2 but they were surrounded by genes encoding extracellular matrix (ECM) components (e.g., laminin subunits and collagen chains), the ITGB6 integrin subunit, and LOXL2 encoding an ECM crosslinking enzyme [69] pointing to modification of ECM production and adhesion.

We addressed to what extent GRHL2 regulated gene clusters identified in our conditional MCF7 KO model predicted associations with GRHL2 gene expression in breast cancer patients. We focused on all genes where the control sgRNA gave 0.75 < FC < 1.5 at each time point after GRHL2 KO while both GRHL2 sgRNAs triggered either FC < 0.75 in at least 3 time points (positive correlation with GRHL2) or FC > 1.5 in at least 3 time points (negative correlation with GRHL2). We made use of a cohort of 867 untreated breast cancer patients (MA-867 dataset [59]) and ranked patients in 4 quartiles according to the level of GRHL2 expression. The average expression of predicted negatively correlated and positively correlated gene clusters based on the MCF7 conditional KO model, displayed a significant correlation with GRHL2 expression in the same direction when all patients were treated as one group (Fig. 7b). Moreover, behavior in the MCF7 conditional KO model correctly predicted the correlation of gene clusters with GRHL2 expression when ER positive and ER negative patients were separately tested.

At the individual gene level, Pearson correlation coefficients for association with GRHL2 when all patients of the MA-867 dataset were treated as one group, were in the range − 0.31 < R < 0.29 indicating that associations while in the same orientation were weak. This included *EHF* and *CLDN4* that were subject to promoter binding by GRHL2 and belonged to the positively correlated gene cluster (Figs. 4c, 6a, 7c; Additional file 8: Table S2). We also analyzed the METABRIC data set consisting of RNAseq data of 1904 primary breast tumors. Here, co-expression analysis using cBioPortal showed a significant correlation in the same direction as predicted by the MCF7 conditional KO model for *CLDN4* but not *EHF* (Fig. 7c). For the negatively correlated gene cluster, *TGFBR2* as well as *TGFB1* showed a significant correlation in the same direction in the METABRIC data set, further establishing suppression of TGFß signaling by GRHL2 in breast cancer cells. Notably, the large majority of genes in both clusters did not harbor promoter binding sites, further indicating that regulation at enhancer sites or indirect mechanisms prevailed.

## Discussion

We report genome-wide binding sites of the transcription factor GRHL2 that are conserved across 3 human luminal breast cancer cell lines. The match with previously published binding motifs in other cell types shows conservation of GRHL2-DNA interaction but we find that the spectrum of GRHL2 targets differs considerably from those identified in other cells. A limited number of binding sites were located at gene promoter regions. Similar to previous reports [14, 17], most binding sites were located in introns and intergenic regions. Such regions may contain enhancers interacting with GRHL2 and GRHL2 has been reported to regulate histone modifications such as H3K4me3 and H3K4me1 [17, 70]. Notably, GRHL2 can regulate ER⍺ signaling output in hormone receptor positive breast cancer by co-occupying enhancer elements with FOXA1, GATA3, and ER⍺ [34–36]. Co-occupation of enhancers by ER⍺ and GRHL2 has been shown to be regulated by ER⍺ phosphorylation at Ser118 [57]. Indeed, we detect an ER⍺ binding motif in the vicinity of GRHL2 peaks, but this represents only a minor fraction of the identified GRHL2 binding sites. Moreover, intersection of our identified GRHL2 peaks with published ER⍺ binding events in the same series of luminal breast cancer cells cultured under the same conditions further indicates that GRHL2 binds most of the targets found by us in absence of ER⍺, FOXA1, and GATA3. A study intersecting binding sites for GRHL2, FOXA1, and ER⍺ in MCF7 cells also found that most GRHL2 binding sites did not overlap with FOXA1 or ER⍺ binding but ~ 30% did show overlap [71]. Our exclusive focus on GRHL2 binding sites that are conserved across three luminal breast cancer cell lines may have selected for those sites binding only GRHL2. Together, these studies indicate that enhancers occupied by ER⍺, FOXA1, and GATA3 frequently also bind GRHL2, but a majority of conserved GRHL2 binding sites in luminal breast cancer cells do not overlap with binding of the ER⍺ signaling complex.

Using a conditional KO model, we identify genes whose transcription is regulated by GRHL2 in luminal breast cancer cells. Notably, the gene clusters showing up- or downregulation in response to GRHL2 loss show a significant, albeit low level of correlation with GRHL2 expression in breast cancer patients. By using Bru-seq we focus on changes in the rate of nascent RNA synthesis caused by GRHL2 depletion [72]. Differences with studies using steady state RNA-seq may be due to post transcriptional mechanisms of regulation not addressed in our analysis, including RNA stability. We observed diverse responses to GRHL2 depletion, including enhanced or repressed transcription that can be sustained, transient or dynamic. The fact that patterns of transcription induction are similar to the patterns of transcription repression is in line with the fact that GRHL2 has been reported to act as a positive as well as a negative regulator of gene transcription. However, indirect mechanisms involving other transcriptional activators or repressors may also be triggered by GRHL2 depletion.

GRHL2 expression appears to support cancer growth and even disease progression in most tumor types investigated [18–22, 37]. Indeed, GRHL2 drives expression of several genes promoting cell survival and proliferation [9, 18, 19]. Our study agrees with this as GRHL2 loss rapidly affects a cluster of genes involved in cell cycle progression and causes a gradual decrease in proliferation in MCF7 cells. A group of genes whose transcription is reduced following loss of GRHL2 is involved in cell cycle progression and DNA replication including the epithelial specific ETS family transcription factor *EHF,* E2F transcription factors *E2F1* and *E2F2,* and other genes such as *CDCA7L* and *MCM2* [60–63]. We show that *EHF, E2F2* and *CDCA7L* represent previously unidentified GRHL2 target genes that can be subject to direct regulation at promotor regions. *EHF* has been previously implicated in ovarian, gastric and prostate cancer [73–75] but our findings point to cooperative roles of GRHL2 target genes including EHF and E2Fs in sustaining proliferation.

Several studies have shown that GRHL2 suppresses EMT [9, 17, 23, 24, 26, 27]. This may explain its reported role as a suppressor of cancer tissue invasion and metastasis [9, 25]. In fact, a similar function may also be involved in the many examples where GRHL2 is positively associated with tumor progression and metastasis. GRHL2 may prevent a complete EMT and maintain cancer cells in a hybrid EMT state that is believed to be crucial for cancer cell plasticity, which supports invasion and metastasis [76, 77]. Our results concerning GRHL2 interactions with known EMT-related genes are partly in disagreement with previously published findings. First, we demonstrate that *CDH1* RNA synthesis is not altered following GRHL2 loss, despite an intronic binding site that is conserved in the three luminal cell lines. No binding site is observed in the − 1000/+ 100 promotor region but we detect GRHL2 binding in the region from − 6000 to − 1000 bp relative to the TSS of the *CDH1* gene, consistent with an earlier study reporting a contact of GRHL2 upstream of the *CDH1* promoter [4]. Although this may facilitate long-distance interactions with the promoter region through chromatin looping [4], loss of this interaction, nor that at the intronic GRHL2 binding site, causes a reduction in *CDH1* transcription in the first 16 days after GRHL2 deletion in our study. Our findings do not rule out CDH1 regulation through indirect, post transcriptional mechanisms including RNA stability that are not measured in Bru-seq and may underlie findings in studies using RNA-seq or PCR analyses, or at the level of translation. Second, it has been reported that *ZEB1* is regulated by GRHL2 directly and, vice versa, that ZEB1 regulates *GRHL2* in a balance between EMT and MET [9, 20, 23, 24]. We do not detect GRHL2 binding sites in the promoter, or other regions of the *ZEB1* or *ZEB2* genes. This potential discrepancy cannot be explained by technical differences as we have confirmed the lack of GRHL2 binding in the ChIP-seq analysis by ChIP-qPCR using primers that amplified ZEB1 and ZEB2 regions bound by GRHL2 in human mammary epithelial cells and human ovarian cancer cells in other studies [17, 23]. Rather, this may point to differences in GRHL2 interactions in different cell types. Nevertheless, the fact that we do not detect GHRL2-binding sites in ZEB1 or ZEB2 is in line with our Bru-seq analysis indicating that transcription of the *ZEB1* and *ZEB2* genes is not affected by GRHL2 depletion in the first 16 days. Together, this data indicates that *CDH1*, *ZEB1*, and *ZEB2* genes do not represent direct transcriptional targets of GRHL2 in luminal breast cancer and their regulation may occur through post-transcriptional regulation in this cellular context. Our data do confirm *CLDN4* as a direct target gene with GRHL2-binding in the promoter region and transcriptional suppression in response to GRHL2 depletion in luminal breast cancer cells.

The fact that in our study GRHL2 supports gene networks involved in cell proliferation and that a tumor/metastasis suppressing function related to its suppression of EMT is less evident, agrees with the location of GRHL2 on chromosome 8q22, a region that is amplified in various cancers, including breast cancer. One explanation for the discrepancy between different studies including our own is the possibility that GRHL2 interacts with- and regulates genes in a context-dependent manner. A meta-analysis combining all RNA-seq, micro-array, and ChIP-seq experiments, identified common candidate genes for regulation by GRH or GRHL1-3. The authors noticed a striking lack of correlation between findings in normal epithelia as compared to cancerous cells with *CDH1* being identified as a target in normal epithelia but not cancer [78]. Likewise, the findings reported in our study represent candidate GRHL2-regulated genes and pathways in luminal breast cancer that partly overlap but are also distinct from GRHL2 regulation in normal epithelia and other cancer types.

## Conclusion

Taken together, this study provides a comprehensive genome-wide resource of GRHL2 binding sites conserved across luminal breast cancer cells. In a conditional KO model, we identify groups of genes whose transcription is positively or negatively controlled by GRHL2 and find 5 main patterns of dynamic regulation. The association with GRHL2 of gene clusters in the KO model predicts the correlation with GRHL2 expression in breast cancer patients. The dominant response to GRHL2 depletion in luminal breast cancer cells is suppression of proliferation and we identify clusters of genes reflecting this response including direct regulation of ETS and E2F transcription factors by GRHL2. An EMT response to GRHL2 loss is limited and our findings indicate that regulation of epithelial genes can be strikingly different in normal and cancer cells involving direct GRHL2-mediated transcriptional control or indirect mechanisms.

## Supplementary Information

**Additional file 1: Fig. S1.** DNA fragmentation analysis by agarose gel electrophoresis. After sonication, indicated samples were purified and loaded on 2% agarose gel.

**Additional file 2: Fig. S2.** ChIP-qPCR validation of the isolated genomic DNA fragments. Graphs represent the efficiency of CLDN4 genomic DNA co-precipitation with anti-GRHL2 Ab (black bars) or IgG control Ab (grey bars). Detection was performed by qPCR using primers targeting the promoter region of CLDN4 or targeting the intergenic region upstream of the GAPDH locus (Control). Results are shown for 3 GRHL2-positive luminal cell lines (MCF7, BT474 and T47D) and 1 GRHL2-negative basal-B cell line (Hs578T).

**Additional file 3: Fig. S3.** Cumulative presence of adapter sequences. Results show that cumulative presence of adapter sequences is less than 5% in each cell sample, indicating that the data sets could be further analyzed without adapter-trimming.

**Additional file 4: Fig. S4.** Per base sequence quality for all sequencing data sets. Y axis is divided into high quality calls (green), reasonable quality calls (orange) and poor-quality calls (red). Analysis shows that the mean quality of base calls, indicated by the blue line, consistently remained in the green area, indicating that sequencing data sets were of high quality.

**Additional file 5: Fig. S5.** Coverage of peak regions across chromosomes. Graphs represent the coverage of GRHL2 binding sites across all chromosomes in the indicated cell lines.

**Additional file 6: Table. S1.** Candidate GRHL2 target genes in luminal breast cancer cells displaying promoter interaction. GRHL2 promotor interactions identified by ChIP-seq in 3 luminal breast cancer cell lines are listed. Genes also identified by Bru-seq in MCF7 conditional KO model showing up- or downregulation at one or more timepoints in response to GRHL2 loss are indicated in bold.

**Additional file 7: Fig. S6.** Complete Western blots for Figure 3b.

**Additional file 8: Table S2.** GRHL2-regulated genes identified by Bru-seq in MCF7 conditional KO model. AFC for indicated genes at the indicated timepoints (days) post induction of GRHL2 KO identified by Bru-seq in MCF7 conditional KO model and assignment to clusters is shown. Genes also displaying promoter interaction identified by ChIP-seq in MCF7cells are indicated in bold.

## Data Availability

Chip-seq data supporting the results of this article is available at the UCSC Genome Browser [https://genome.ucsc.edu/s/hwuRadboudumc/ZWang]. Bru-seq data supporting the results of this article is available at Gene Expression Omnibus (GEO) database, www.ncbi.nlm.nih.gov/geo (Accession No. GSE222353).

## Abbreviations

GRHL2: Grainyhead like 2
EMT: Epithelial to mesenchymal transition
ER: Estrogen receptor
HER2: Human epidermal growth factor receptor 2
ChIP-seq: Chromatin immunoprecipitation followed by high-throughput DNA sequencing
qPCR: Quantitative polymerase chain reaction
Bru-seq: Bromouridine sequencing
RIPA buffer: Radioimmunoprecipitation buffer
BCA: Bicinchoninic acid
PVDF: Polyvinylidene difluoride
BSA: Bovine serum albumin
RT: Room temperature
IPA: Ingenuity pathways analysis
DAVID: Database for annotation, visualization, and integrated discovery
GO: Gene ontology
PANTHER: Protein analysis through evolutionary relationships
AFC: Average fold change
PPI: Protein–protein interaction
