# Supplementary figures and images for: GRHL2-controlled gene expression networks in luminal breast cancer
Source: Cell Commun Signal. 2023 Jan 23;21:15. doi: 10.1186/s12964-022-01029-5 (PMC9869538; doi:10.1186/s12964-022-01029-5)

Fig. S1

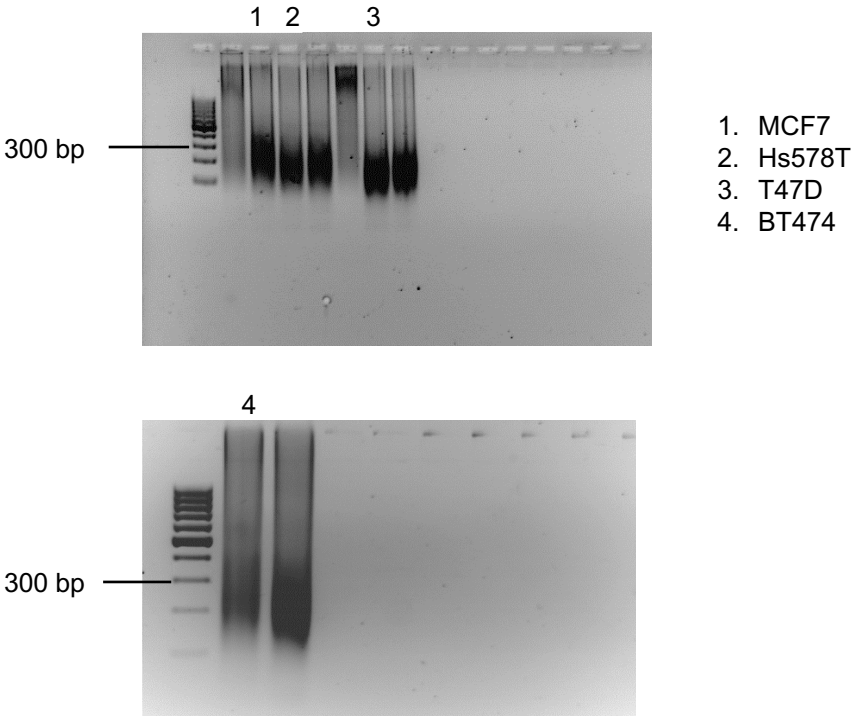

Supplement: Supplementary file 2 — Additional file 1: Fig. S1. DNA fragmentation analysis by agarose gel electrophoresis. After sonication, indicated samples were purified and loaded on 2% agarose gel. [file 12964_2022_1029_MOESM2_ESM.pdf]

Fig. S2

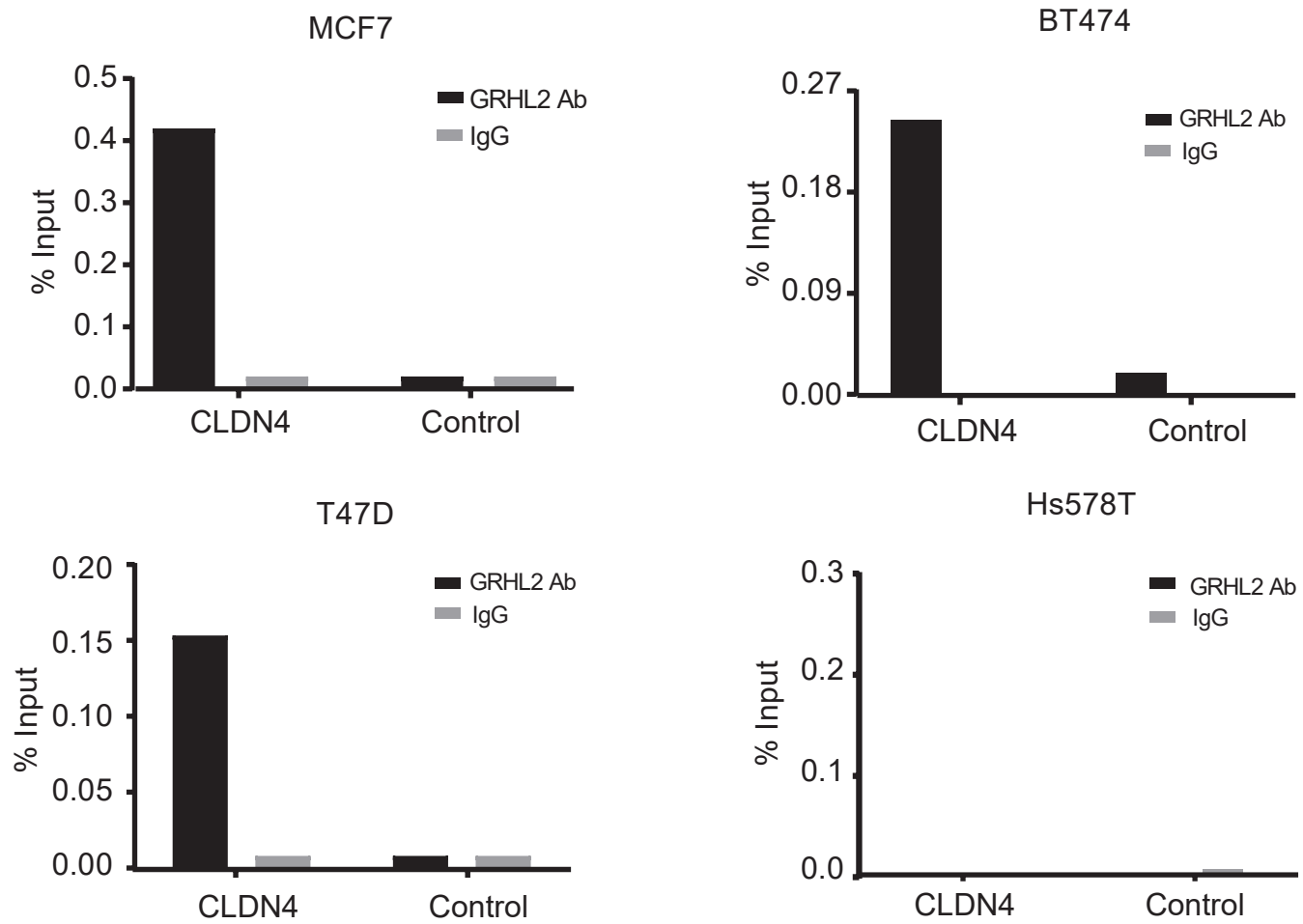

Supplement: Supplementary file 3 — Additional file 2: Fig. S2. ChIP-qPCR validation of the isolated genomic DNA fragments. Graphs represent the efficiency of CLDN4 genomic DNA co-precipitation with anti-GRHL2 Ab (black bars) or IgG control Ab (grey bars). Detection was performed by qPCR using primers targeting the promoter region of CLDN4 or targeting the intergenic region upstream of the GAPDH locus (Control). Results are shown for 3 GRHL2-positive luminal cell lines (MCF7, BT474 and T47D) and 1 GRHL2-negative basal-B cell line (Hs578T). [file 12964_2022_1029_MOESM3_ESM.pdf]

Fig. S3

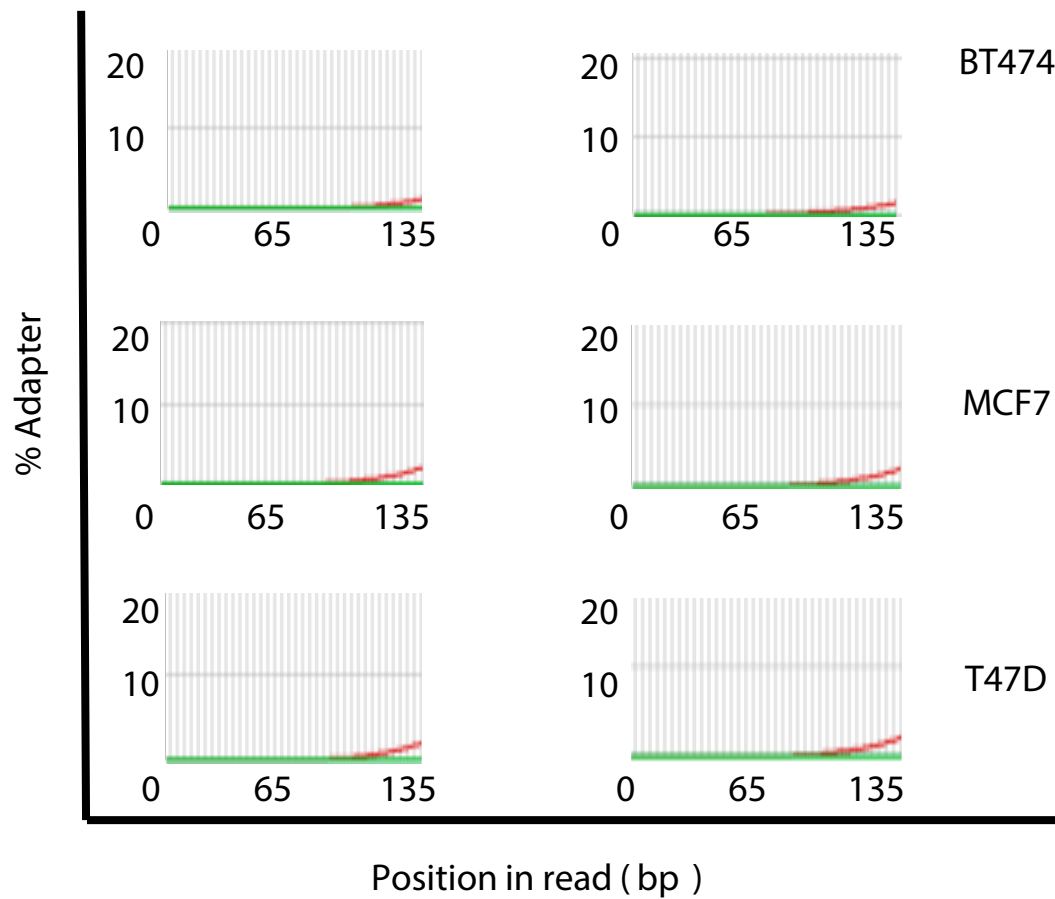

Supplement: Supplementary file 4 — Additional file 3: Fig. S3. Cumulative presence of adapter sequences. Results show that cumulative presence of adapter sequences is less than 5% in each cell sample, indicating that the data sets could be further analyzed without adapter-trimming. [file 12964_2022_1029_MOESM4_ESM.pdf]

Fig. S4

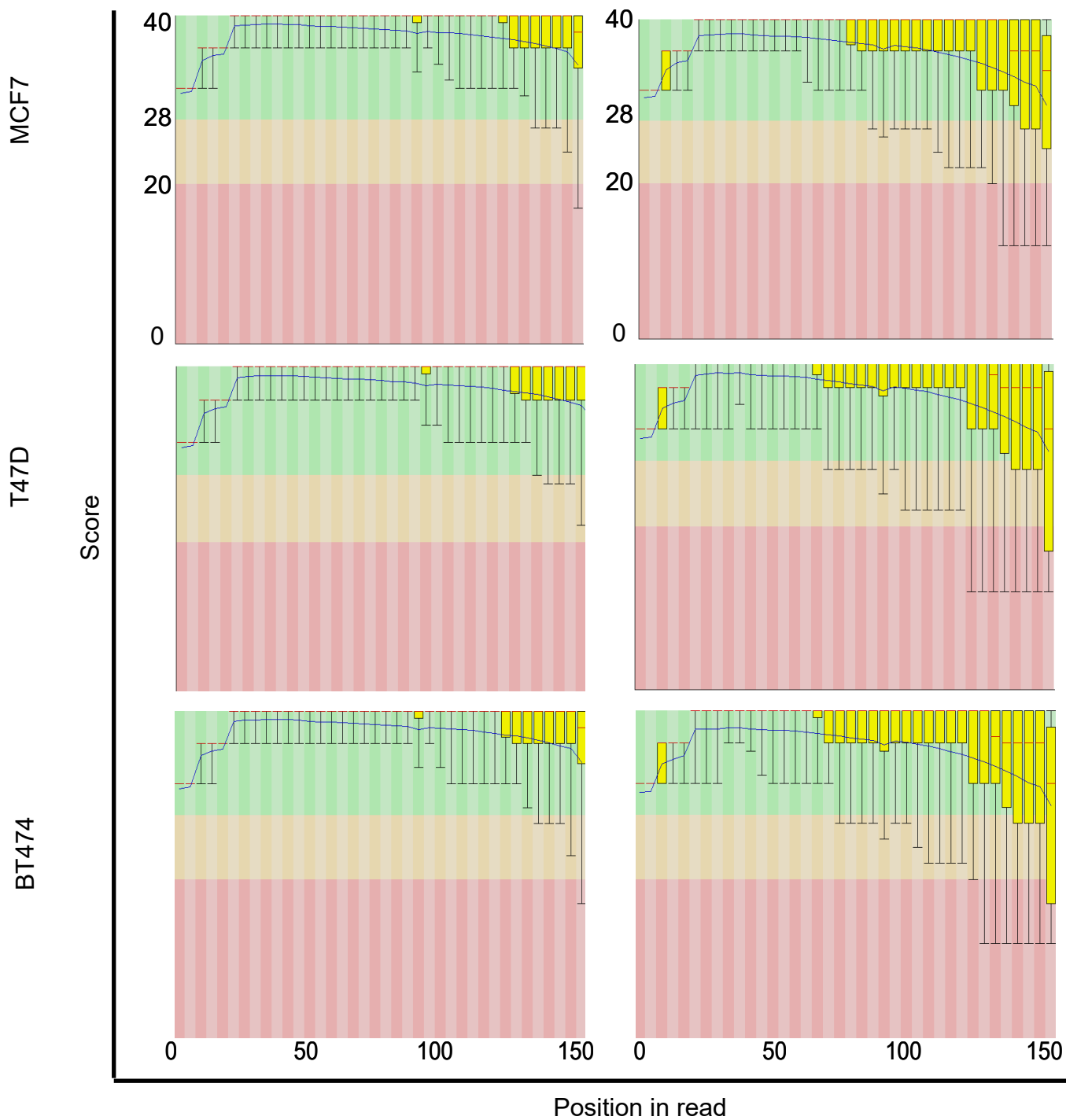

Supplement: Supplementary file 5 — Additional file 4: Fig. S4. Per base sequence quality for all sequencing data sets. Y axis is divided into high quality calls (green), reasonable quality calls (orange) and poor-quality calls (red). Analysis shows that the mean quality of base calls, indicated by the blue line, consistently remained in the green area, indicating that sequencing data sets were of high quality. [file 12964_2022_1029_MOESM5_ESM.pdf]

Fig. S5

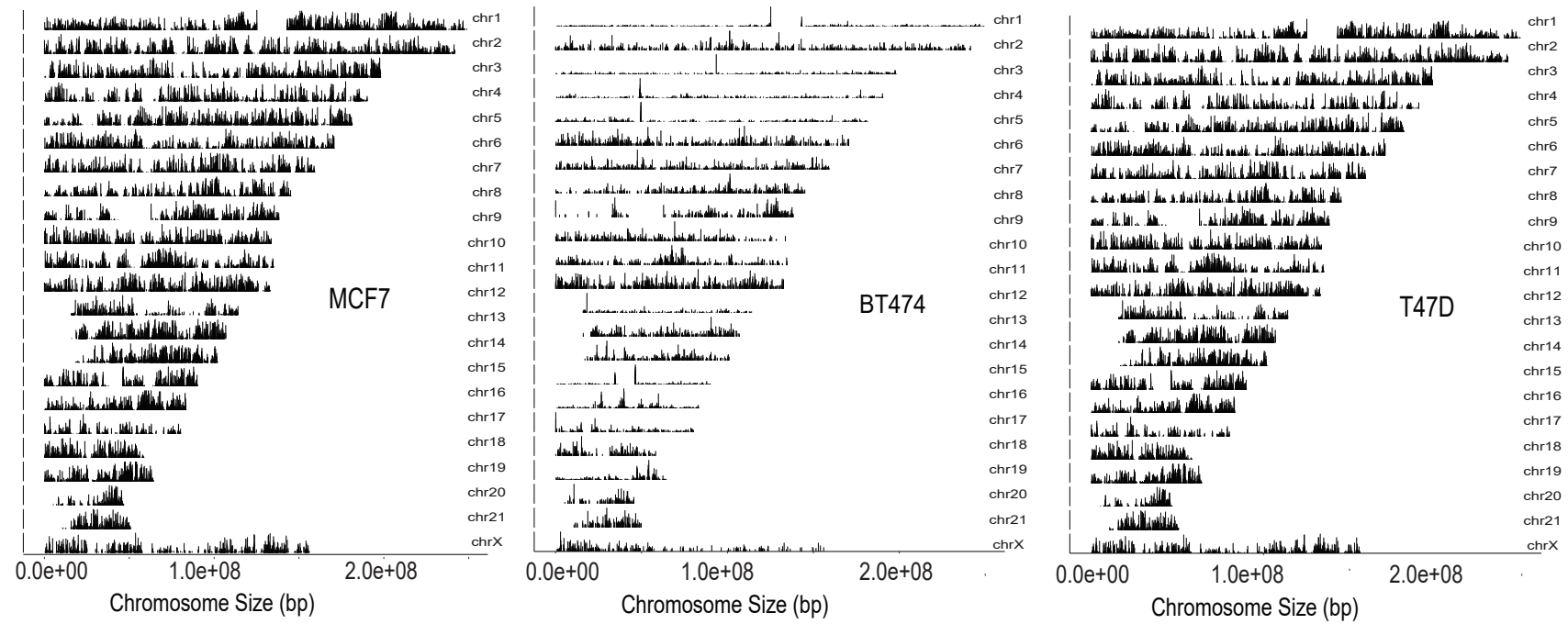

Supplement: Supplementary file 6 — Additional file 5: Fig. S5. Coverage of peak regions across chromosomes. Graphs represent the coverage of GRHL2 binding sites across all chromosomes in the indicated cell lines. [file 12964_2022_1029_MOESM6_ESM.pdf]
