# Supplementary material for: GRHL2-controlled gene expression networks in luminal breast cancer
Source: Cell Commun Signal. 2023 Jan 23;21:15. doi: 10.1186/s12964-022-01029-5 (PMC9869538; doi:10.1186/s12964-022-01029-5)
Supplement: Supplementary file 8 — Additional file 7: Fig. S6. Complete Western blots for Figure 3b. [file 12964_2022_1029_MOESM8_ESM.pdf]

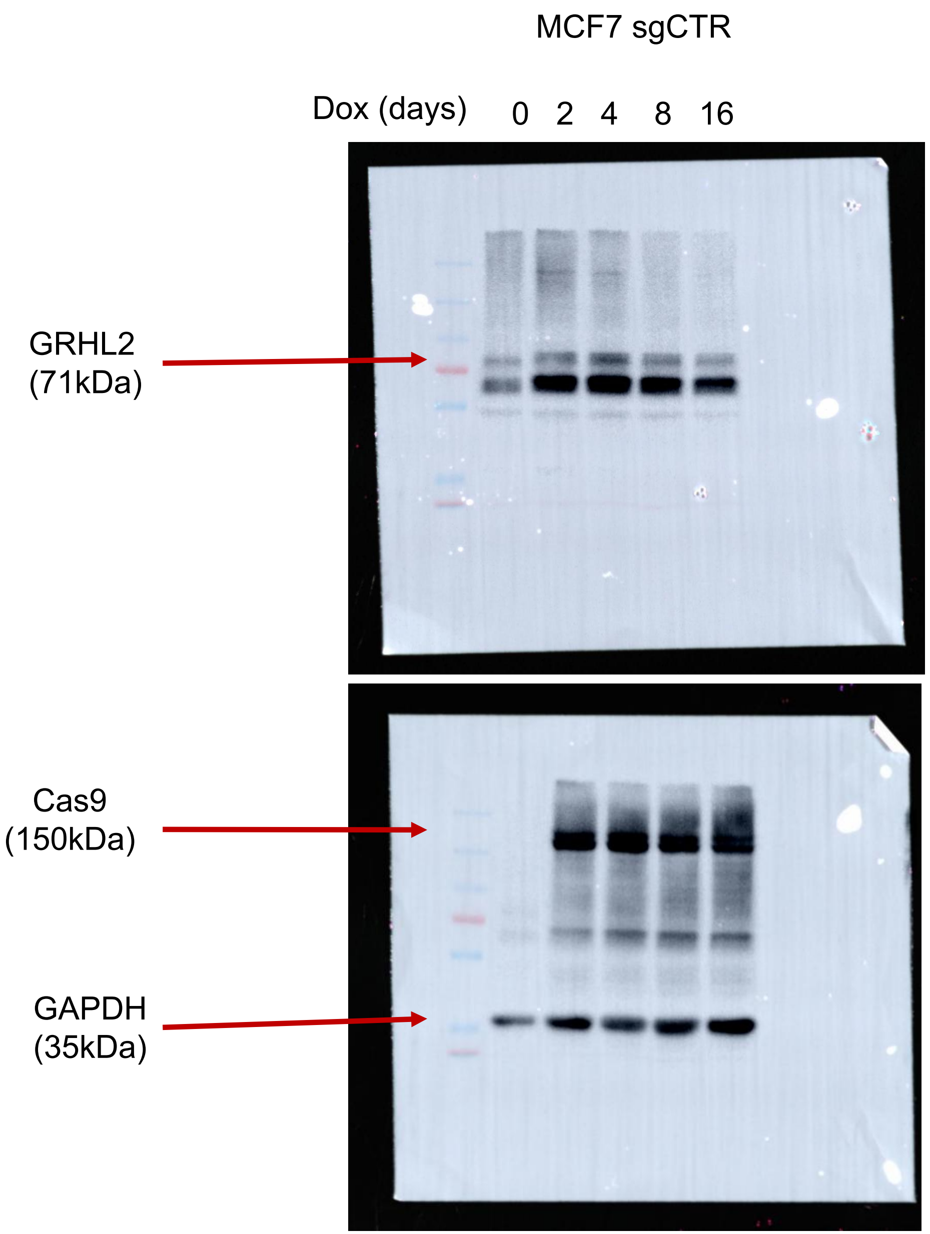
MCF7 sgCTR
Dox (days)
0
2
4
8
16
GRHL2
(71kDa)
Cas9
(150kDa)
GAPDH
(35kDa)

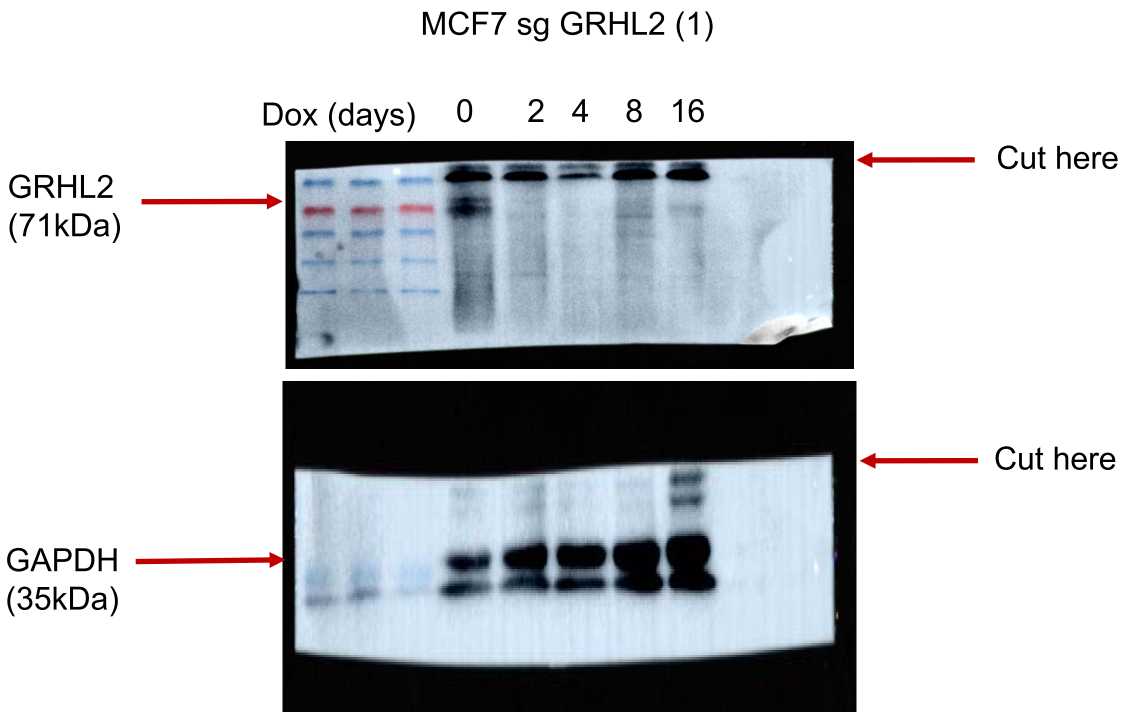
MCF7 sg GRHL2 (1)
Dox (days) 0 2 4 8 16
GRHL2
(71kDa)
Cut here
GAPDH
(35kDa)
Cut here

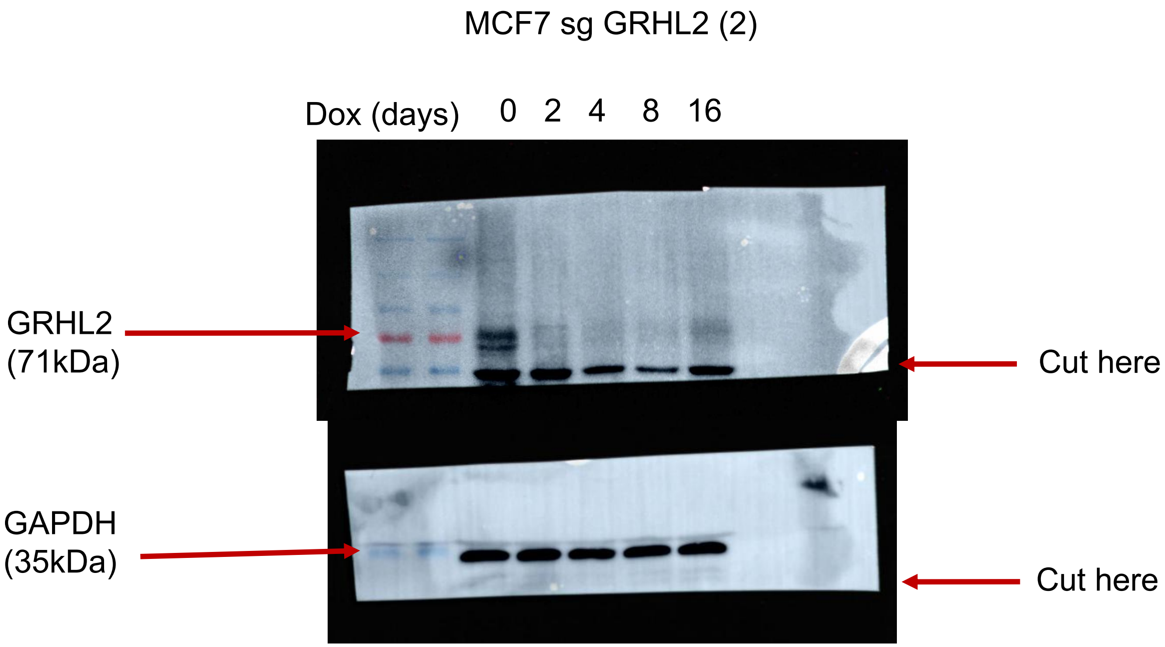
MCF7 sg GRHL2 (2)
Dox (days)
0
2
4
8
16
GRHL2
(71kDa)
Cut here
GAPDH
(35kDa)
Cut here
